# Opening the door to university health research: recommendations for increasing accessibility for individuals with intellectual disability

**DOI:** 10.1186/s12939-022-01730-4

**Published:** 2022-09-10

**Authors:** Brittany M. St. John, Emily Hickey, Edward Kastern, Chad Russell, Tina Russell, Ashley Mathy, Brogan Peterson, Don Wigington, Casey Pellien, Allison Caudill, Libby Hladik, Karla K. Ausderau

**Affiliations:** 1 Department of Kinesiology, Occupational Therapy Program, 2120 Medical Sciences Center, University of Wisconsin at Madison, WI 53706, 1300 University Avenue, Madison, WI USA; 2 Waisman Center, University of Wisconsin, Madison, WI USA; 3 Special Olympics Wisconsin, Madison, WI USA; 4 Institutional Review Board, University of Wisconsin at Madison, Madison, WI USA

**Keywords:** Intellectual disability, Health research, Accessibility, Inclusive, Research, Recommendations

## Abstract

**Background:**

Advances in health equity rely on representation of diverse groups in population health research samples. Despite progress in the diversification of research samples, continued expansion to include systematically excluded groups is needed to address health inequities. One such group that is infrequently represented in population health research are adults with intellectual disability. Individuals with intellectual disability experience pervasive health disparities. Representation in population health research is crucial to determine the root causes of inequity, understand the health of diverse populations, and address health disparities. The purpose of this paper was to develop recommendations for researchers to increase the accessibility of university health research and to support the inclusion of adults with intellectual disability as participants in health research.

**Methods:**

A comprehensive literature review, consultation with the university ethics review board, and review of United States federal regulations was completed to identify barriers to research participation for individuals with intellectual disability. A collaborative stakeholder working group developed recommendations and products to increase the accessibility of university research for participants with intellectual disability.

**Results:**

Eleven key barriers to research participation were identified including gaps in researchers’ knowledge, lack of trust, accessibility and communication challenges, and systematic exclusion among others. Together the stakeholder working group compiled seven general recommendations for university health researchers to guide inclusion efforts. Recommendations included: 1) address the knowledge gap, 2) build community partnerships, 3) use plain language, 4) simplify consent and assent processes, 5) establish research capacity to consent, 6) offer universal supports and accommodations, and 7) practice accessible dissemination. In addition, four products were created as part of the stakeholder working group to be shared with researchers to support the inclusion of participants with intellectual disability. 1) Supports I Need Checklist, 2) Plain language glossary of health and research terms, 3) Understanding Consent and Assent in Plain Language, 4) Easy-Read Paper Template.

**Conclusion:**

Community members and individuals with intellectual disability want to be included in research and are eager to engage as research participants. It is the responsibility of the researcher to open the door to university health research. The recommendations discussed in this paper could increase accessibility for a broader range of research participants and, in particular, promote the inclusion of individuals with intellectual disability to advance health equity in population health research.

**Supplementary Information:**

The online version contains supplementary material available at 10.1186/s12939-022-01730-4.

## Background

Advances in health equity rely on representation of diverse groups in health research samples. Sample demographics have diversified over time, and National Institutes of Health regulations in the United States have evolved to require women and minorities be included as participants in clinical research [1] and to further consider inclusion across the lifespan [2]. However, despite progress, population health research and clinical research samples have continued to be critiqued as falling short of representation of the general population [3, 4]. A concerted effort to include systematically excluded groups is needed to characterize and address health inequities. One such group that is infrequently represented in research are adults with intellectual disability [5, 6].

Intellectual disability is a lifelong neurodevelopmental condition beginning in childhood that is characterized by cognitive differences and adaptive functioning challenges in conceptual, social, and practical domains [7]. Intellectual disability comes from a variety of etiologies including genetic conditions, illness, and injury so long as impairments begin during the developmental period [7]. Individuals with intellectual disability are often represented within a broader designation of individuals with intellectual and developmental disabilities. However, intellectual disability is an important distinction from intellectual and developmental disability. The classification of intellectual and developmental disability includes a broad group of conditions that begin in early childhood and have impacts on physical, learning, language, or behavior areas [8] whereas intellectual disability is a separate condition with defined diagnostic criteria as described above [7].

Prevalence of intellectual disability has proven difficult to estimate due to the lack of surveillance and health data available [9]. Two recent systematic reviews on the prevalence of intellectual disability in the United States [10] and internationally [11] have identified a wide range of estimated prevalence rates (0.05 to 1.55% of the population) depending on age range and data source [10, 11]. Rates across reviewed studies are not directly comparable due to differences in age of the samples, thereby complicating any consistent prevalence estimate and highlighting the need for added health surveillance for individuals with intellectual disability [10, 11]. Individuals with intellectual disability have higher support needs and greater levels of healthcare use [12, 13], further supporting the importance of including them in research designing and evaluating the effectiveness of health interventions. Approximately 14% of all working-age Supplemental Security Income and Social Security Disability Insurance beneficiaries in the United States are individuals with intellectual disability [12]. The increased representation of individuals with intellectual disability among social insurance beneficiaries may indicate increased service need or usage. Representation in population health samples and targeted research samples is crucial to develop a comprehensive understanding of the unique needs of individuals with intellectual disability and opportunities for targeted support and interventions.

Adults with intellectual disability experience pervasive and well documented health disparities. Multiple studies globally have identified elevated rates of chronic conditions such as asthma, epilepsy, and diabetes [13–16] in individuals with intellectual disability. Further, studies have identified disparities in healthcare quality and access for individuals with intellectual disability [17–19]. A large study in Scotland, for example, identified that long-term conditions were more poorly managed for individuals with intellectual disability across 66.7% of indicators (e.g., % of participants receiving screening, testing and treatments for common chronic health conditions within recommended timeframes) in comparison to the general population [15]. One of the most concerning health disparities documented for individuals with intellectual disability is a significantly increased risk of early mortality [20–26]. Several studies have identified that not only are individuals with intellectual disability more likely to die earlier, they experience higher rates of potentially avoidable deaths [20, 22–24]. Potentially avoidable deaths are those from conditions that are treatable or preventable within the current healthcare systems for people under the age of 75 (e.g., most infections) [24]. Individuals with intellectual disability have complex health needs and evidence-based information on their health and health management is needed [16]. Together, this literature highlights the complex health needs of individuals with intellectual disabilities and suggests that evidence-based information on their health and health management is needed to address disparities in healthcare quality and access.

Univeristy health research in the fields of public health and clinical research has documented that the exclusion of individuals with intellectual disability is pervasive in their respective fields [4–6, 27, 28]. A recent conference paper presented findings from a review of inclusion and exclusion criteria of US-based NIH-Funded clinical trials with start dates between 2018 and 2021 to identify to what extent adults with intellectual disability are excluded from participation [28]. Schwartz and collegues reviewed 248 trials and identified 74.6% of studies likely excluded adults with intellectual disability through criteria including ability to read and write, functional capacity, and access to technology among others [28]. Further, 32.6% of the studies reviewed directly excluded adults with intellectual disability through consent capacity requirements and exclusion based on cognitive capacity or diagnosis. Previous literature demonstrates exclusion of individuals with intellectual disability has been an identified and pervasive issue in public health and clinical research. A systematic review of articles published in public health journals found exclusion of intellectual disability in 72% of the studies reviewed [6]. Specifically, reviewed cohort studies demonstrated passive exclusion of individuals with intellectual disability and randomized control trials displayed active exclusion of individuals with intellectual disability [6]. An additional review of 300 randomly chosen clinical trial studies published in six high impact medical journals identified over 90% of the reviewed study designs explicitly excluded (did not allow to participate in the study) or likely excluded individuals with intellectual disability from participating based on the study exclusion criteria [5]. Specific exclusion criteria including neurological and cognitive disturbances, physical disabilities, presumed poor adherence, exclusion by score on assessment such as the Mini-Mental Status Examination, communication deficits, and requirement for written informed consent contributed to either explicit or likely exclusion of individuals with intellectual disability [5]. Only 6 of the 300 studies reviewed identified individuals with intellectual disability in their samples [5]. Even within broader neurodevelopmental disability studies, individuals with intellectual disability are vastly underrepresented. A recent meta-analysis of studies in high impact autism specific journals found that of 100,245 autistic participants, 94% did not have co-occurring intellectual disability [27]. Population level estimates of autistic individuals without intellectual disability are approximately 67% [29], highlighting likely selection bias against intellectual disability even within disability specific research. In addition to bias exclusion criteria, individuals with intellectual disability face a wide range of barriers to participation.

Individuals with intellectual disability are motivated to participate in research [30] yet barriers to participation often impede their ability to do so. One such well documented barrier is the perception that adults with intellectual disability lack the capacity to consent, leading to explicit exclusion of potential participants without assessing capacity in a meaningful way [28, 31]. Adaptations to the consent process to increase accessibility or allow for consent through a leagally authorized representative or guardian are infrequently offered [28, 32, 33]. In addition, research recruitment leading to participation may need to be facilitated by a service provider, guardian, or other legally authorized representative. Adults with intellectual disability may not be exposed to recruitment information or be aware of research opportunities if researchers are not altering recruitment and enrollment procedures to include additional support or decision making personnel [30, 34]. Adaptations to procedures, language, and accessibility of research spaces may be necessary for participation and are infrequently available to participants [4, 28, 35, 36]. Identification of additional barriers to participation in research for individuals with intellectual disability is an important first step toward identifying solutions to address barriers and increase representation in university health research.

The current paper is focused on expanding accessibility of university health research for the inclusion of adults with intellectual disability as research participants. Inclusion of individuals with intellectual disability as research participants is a vital first step to advance representation within research samples and identification of modifiable mechanisms contributing to health disparities. However, of equal importance is the expansion of inclusive community engaged research practices that bring in co-researchers with intellectual disability throughout the research process. Thus, recommendations for next steps and expansion from this work to effectively incorporate co-researchers with intellectual disability throughout each stage of the research process, are presented in the discussion section.

### Purpose

The purpose of this paper was to work with co-researchers with intellectual disability to develop recommendations for university researchers to increase the accessibility of university health research and to support the inclusion of adults with intellectual disability as research participants.

## Methods

The current work used a community-engaged research design and applied the design principles for stakeholder engagement presented by Boaz and colleagues [37]. Self-advocates with intellectual disability, family members of individuals with intellectual disability, representatives from community organizations, and institutional review board staff served as collaborative partners throughout the research process. Recommendations to increase the accessibility of university research for adult (i.e., 18 years of age or older) participants with intellectual disability were developed following a comprehensive literature review, consultation with the institutional review board, detailed review of the federal regulations applicable to university research in the United States, and the creation of summaries and products developed with a stakeholder working group.

### Previous work and project development

Our research program has established a foundation of over 6 years of continuous stakeholder engaged research with self-advocates with intellectual disability, family members of individuals with intellectual disability, and community organizations that serve individuals with intellectual disability [38, 39]. Community engagement was prioritized across all previous projects, yet barriers to inclusion of individuals with intellectual disability were frequently identified by researchers and self-advocates. Based on the experience of researchers working within the confines of a university institution and collaborative experiences with stakeholders, it was determined that there was a need for a comprehensive review of the accessibility of university research. Based on the review, there was a clear need for the development of recommendations and tools to improve the accessibility of research for adult participants with intellectual disability. This work directly informed and led to the current project. Recommendations were collaboratively developed with a stakeholder working group following the literature review, institutional ethics review board consultation, and review of applicable federal regulations.

### Literature review of barriers to inclusion of individuals with intellectual disability

An initial review of the literature published in the last decade was completed to determine what current barriers to inclusion of adult participants with intellectual disability had been identified by previous research. A team of four graduate students searched article databases for published journal articles from any field that focused on the participation of adults with intellectual disability as research participants and co-researching partners. Databases searched included PubMed, CINHAL, and Google Scholar using a variety of key search terms (e.g., intellectual disability, research participant, co-researcher, inclusion, university research, health research, medical research, and selection bias). Articles were reviewed for identified barriers to participation for individuals with intellectual disability. All barriers identified through the literature review process were compiled into a detailed list for use in the next phases of this project.

### Consultation with the institutional review board (IRB)

Two research team members met with a representative of the IRB to discuss documents and policies related to the inclusion of adults with intellectual disability in research. The objective of the consultation was to understand how researchers could better partner with university institutions to ethically and effectively include individuals with intellectual disability in health research. The primary areas of discussion were how consent capacity is determined, increasing the accessibility of the consent process, discussion on the language used by the IRB in policies and documents, self-advocate response to current language used, and opportunities for adapted or supported human subjects research training for research staff members and co-researchers with intellectual disability. The discussion was informed by an initial review of institutional Human Research Protection Program policies and procedures, previous experience in obtaining approval for research including individuals with intellectual disability, and barriers identified from the literature to research participation relevant to the ethics review board’s approval.

### Review of United States Federal Regulations for university research

A comprehensive review of United States federal regulation documents applicable to university research was completed. Two student research assistants identified relevant documents and compiled them for review. Each document was reviewed to answer two key questions: 1) Does this document specifically mention research with individuals with intellectual disability or research with vulnerable populations? 2) How does this document guide or support the inclusion of individuals with intellectual disability?

### Stakeholder working group

A stakeholder working group (“Opening the Door Working Group”) was assembled with four self-advocates with intellectual disability, two parents of an adult child with intellectual disability, one representative from a community partner organization, and two university researchers. The working group was created to support the Health Research Summit, a two-day virtual conference. The Health Research Summit provided an opportunity for collaborative information sharing and discussion to continue to work toward building sustainable communities of stakeholders to support ongoing research partnerships to address stakeholder driven health priorities. The Summit theme was increasing health research access and engagement among self-advocates with intellectual and developmental disabilities, family members, community support organizations and university health researchers. The Opening the Door Working Group collaboratively established key objectives for their work together to prepare for the Health Research Summit. Three primary objectives for the group were developed: 1) review barriers identified through comprehensive literature review, institutional ethics review board consultation and review of United States federal regulations, 2) build recommendations based on identified barriers, and 3) generate products to increase accessibility and inclusion of university research.

Working group members met virtually four times over 2 months leading up to participation in the Ausderau Lab virtual Health Research Summit. Working group meetings were 1–1.5 hours long. Group objectives were reviewed during the first meeting and one research member facilitated all four meetings based on a group developed agenda. All members of the working group contributed to the development of recommendations and ideas. All working group members participated in the virtual Health Research Summit over the 2 days including a 2-hour session dedicated to the topic of increased accessibility for university research. Working group members hosted breakout discussions with Health-research Summit attendees. Each breakout session reviewed a developed product and asked attendees to discuss “what is missing?” and “what other factors of accessibility need to be considered for this product?” Working group members reconvened after the Health Summit to review feedback on developed products and plan for dissemination. Self-advocate and community partner members provided review, feedback, and final approval on the final version of all developed products and are contributing authors on this manuscript.

## Results

Data from all sources was integrated into a list of 11 primary barriers to participation in university research for adults with intellectual disability. The barriers to research participation for individuals with intellectual disability were identified through review of previous literature, review of United States federal regulations, consultation with the ethics review board and stakeholder working group process. Barriers to research participation spanned researchers’ individual skills and knowledge, communication and language barriers in research materials and processes, systemic barriers to participation, environmental accessibility, and gaps in accommodations for the unique needs and capabilities of individuals with intellectual disability. A summary of identified barriers to research participation for individuals with intellectual disability are presented in Table 1. The literature identifying these barriers has been cited within the table and integrated into the references section of this paper.

**Table 1:** Barriers to Research Participation for Individuals with Intellectual Disability

Identified Barrier	Description
Gaps in researchers’ skills or knowledge	• Researchers may lack knowledge about the capabilities of people with intellectual disability or how to include people with intellectual disability in their research [40, 41] •Self-advocates have reported a sense of disrespect from the researcher community and a desire for more respectful interactions [40, 42] •Researchers have stereotypes about people with disabilities and their ability to participate in research activities [40, 41]
Lack of Trust in the Research or Research Staff	•People may have difficulty trusting a researcher who does not have an established relationship with a trusted community partners [40] •Individuals may be concerned the research may be used in a way that the individual does not approve of [41] •Trust in the researcher is a critical component to successful participation in research [30, 43]
Environmental Accessibility	•Limited accessibility of the study facility [4, 40, 41] •Scheduling of research activities can be difficult, especially when researchers’ schedules are not flexible enough [40] •Transportation to and from research events can be difficult to arrange or not available [40, 41] •Access to necessary technology (e.g., having internet, computer, phone, or other way to access virtual meetings or events) [40]
Communication	•Challenges with communication between professionals and participants [41] •Non-speaking communication methods may not be accepted or available (e.g., adaptive and alternative communication methods, American Sign Language, non-speaking communication using hand movements and facial expressions) [40]
Inaccessible language and documents	•Self-advocates reported the language used by researchers was hard to understand and too complicated [40, 41] •Research documents, specifically consent forms, are difficult to read and understand [40, 44] •Difficult to balance inclusion of all required information to meet the ethics review board requirements and remain succinct and accessible [45] •Results of studies are not shared with people with disabilities in an accessible way [40, 44]
Use of outdated or offensive language in research policy and documents	•Language used at the institutional level must match language used at the regulatory level (United States federal regulations), despite community awareness of outdated terminology (e.g., “diminished decision-making capacity”) [45]
Challenges with successful recruitment	•People with intellectual disability may not be exposed to recruitment information or know how to find a research study to participate in [30, 34, 40, 46] •Individuals with intellectual disability may need altered recruitment methods that include multiple meetings to feel confident participating [35] •Individuals with intellectual disability may need to be recruited and consented through “gatekeepers” such as service providers and or legal guardians to participate in research [32, 33, 43] •Legal requirement to complete recruitment, consent and assent with an individual’s legal guardian or legally authorized representative can add complexity [47]
Perceived lack of ability to participate in the consent and assent process	•Individuals with intellectual disability are frequently considered to be in a vulnerable category including a “limited capacity to consent” [33, 45, 47] •Individuals with intellectual disability may be *perceived* as lacking the ability to give informed consent [4, 31] •Researchers may not provide the additional time and accommodations that may be needed for ensuring that individuals with intellectual disability understand the purpose and implications of their research participation [32] •Assessment of individuals’ capacity to consent is commonly required by the ethics review board and in some instances, completed through standardized cognitive measures instead of an assessment of their understanding for the specific study [45] •Deficit focus of policy and procedures around the capacity of an individual with an intellectual disability to consent may add additional burden to their participation (e.g., cognitive assessment or multiple consent and assent processes to confirm capacity) [45, 47] •Mismatch in the evaluation of risk and choice about participation between an individual with intellectual disability and their legal guardian or family member [36, 48]
Medical challenges	•Medical problems interfere with participation [41] without accomodations •Difficulty obtaining measurements or completing study procedures [4]
Systematic exclusion from research participation	•Exclusion from participation due to status as a “vulnerable population” according to federal regulations and institutional ethics review board definitions [45, 47] •Study criteria may explicitly exclude the participation of individuals with an intellectual disability [4–6] •Research that includes individuals with intellectual disability is primarily disability focused research [5, 6, 27]
Interest in or agreement with research project aims	•Individuals may be hesitant to join research based on concerns that the results of the research may be used in a way that increases stigma and or causes harm to people with disabilities [41]

*Note:* Barriers were identified through comprehensive literature review, consultation with the institutional review board, review of United States federal regulations relevant to university research, and the Opening the Door Stakeholder Working Group meetings

### Federal regulation review

Policies and procedures that guide human subjects research are essential for the protection of vulnerable populations. This approach to research and protection of vulnerable populations is vital. However, it may contribute to systematic exclusion from health services research participation for individuals with intellectual disabilities in the name of protection. Two primary federal regulations were identified as relevant to the inclusion of individuals with intellectual disability in research, the Belmont Report [49] and the Federal Policy for the Protection of Human Subjects, also known as the Common Rule [50]. The historical context of both these regulations, initially developed in 1979 and 1991 respectively was one of protection in response to significant human rights violations in research. Individuals with intellectual disability are broadly considered to meet the definition of a “vulnerable population” who may have “impaired decision-making capacity” [49, 50].

The designation as vulnerable has implications for how researchers conduct their research as well as additional procedures to ensure participation is not coercive. Implementation of regulations for human subjects research and additional research protections for vulnerable populations was essential. Inadvertently these regulations and protections may contribute to barriers to participation for individuals with intellectual disability. Protective regulations focus on impaired capacity and risk of coercion which has likely contributed to the use of standardized cognitive measures to determine capacity and blanket exclusion of participants with any cognitive limitations to avoid risk. Individuals who are interested in and capable of informed participation in research are likely excluded using these broad measures.

Legal guardianship through appointed leagally authorized representatives can also present challenges to recruitment, enrollment, and inclusion. Protective legally authorized representatives are required to provide consent on behalf of the individual with intellectual disability using “substituted judgement” regardless of the risk level of the study [49]. The implications of this regulatory requirement are such that all communication and consent processes must be completed through the legally authorized representative. This additional step in the enrollment process, while essential and protective, may minimize researchers’ willingness to include individuals with guardians. Inclusive research is not in conflict with protection of vulnerable populations. Additional approaches to successful recruitment, obtaining informed consent, and enrollment processes can maintain the protection of individuals while simultaneously making university research more accessible. Barriers to the inclusion of adults with intellectual disability in health research identified through reviewing United Stated Federal Regulations have been included in Table 1.

### Recommendations to increase the accessibility of university research

Together the stakeholder working group compiled seven general recommendations for university health researchers to guide inclusion efforts. Details for each recommendation are described below and Table 2 provides a list of example practices aligned with each recommendation. In addition, four products were created as part of the stakeholder working group to be shared with researchers to support the inclusion of participants with intellectual disability. 1) Plain language glossary of health and research terms: The glossary is designed to provide a starting place and example for researchers in translating complex documents into plain language. 2) Understanding Consent and Assent in Plain Language: A consent and assent form companion document to support participant understanding of the process. 3) Supports I Need Checklist: The checklist provides a foundation for researchers to provide helpful accommodations, support the inclusion of individuals with diverse needs as participants, and support participants in communicating the accommodations they need. 4) Easy-Read Paper Template: A plain language summary template for academic papers that can be used to disseminate findings to communities and participants. Table 2 connects the stakeholder developed resources with identified recommendations when applicable. All stakeholder created products are available as Additional files.

**Table 2:** Recommendations and Examples for University Researchers to Increase Inclusion of Participants with Intellectual Disability

Recommendation	Examples
1. Address the Knowledge Gap	•Explicitly describe inclusive strategies used to successfully include individuals with intellectual disability •Identify inclusion of individuals with intellectual disability in all study dissemination •Identify the capabilities and contributions of individuals with intellectual disability through dissemination
2. Build Community Partnerships	•Connect with community organizations or providers who serve individuals with intellectual disability •Identify opportunities for mutually beneficial partnerships between research and community stakeholders
3. Use Plain Language	•Simplify language across all research documents and materials •Use a glossary in the document to define complex words that cannot be simplified **Resource:** glossary of plain language definitions for common health and research terms (Additional file 1)
4. Simplify Consent (and Assent) Processes	•Create consent and assent forms that are written in plain language, include simple images or diagrams when possible, and are succinct •All consent and assent forms should be formatted using principles of universal accessibility [51] •Use a companion document (e.g., Additional file 2) to support understanding of the consent and assent process •Include additional stakeholders (e.g., caregivers, trusted friends, family) to support trust and informed consent •Obtain both informed consent and informed assent when applicable **Resource:** Understanding Consent and Assent in Plain Language summary (Additional file 2)
5. Establish Research Capacity to Consent	•Only assess understanding as relevant to the current study •Assess understanding through questions to confirm understanding and informed consent •Avoid the use of standardized cognitive measures (e.g., mini mental status exam) to assess the capacity of a person to provide consent
6. Offer Universal Supports and Adaptations	•Choose research locations that are accessible (e.g., ramps, elevators, accessible bathrooms) •Look for locations with easy access to public transportation (e.g., on a public transit route, available parking, signage) •Offer transportation to participants •Make accommodations available to all participants throughout research procedures •Provide information in multiple formats (accessible printed materials, assistance offered for reading or writing). •Individuals should determine the supports they would like to receive. **Resource:** Supports I Need Checklist (Additional file 3)
7. Practice Accessible Dissemination	•Utilize the Easy-Read paper template to create accessible summaries of published findings •Identify alternative formats that are easily accessed by a broad audience (e.g., newsletters, videos, social media postings, and summaries of academic papers) Identify other outlets for disseminating to community audiences (e.g., community presentations, social media posts and videos) •Include self-advocates and community partners in the dissemination process whenever possible **Resource:** Easy-Read Paper Template (Additional file 4)

#### Recommendation 1: address the knowledge gap

Researchers should work to increase their own and other researchers’ knowledge and awareness of how to include adults with intellectual disability in research. Stakeholders highlighted the importance of respectful and strengths focused inclusion and noted the experience of disrespect or stigma. Stakeholders emphasized how researchers need to focus on the capacities and strengths of individuals with disabilities, offer meaningful ways for individuals to contribute to research, and value inclusive research. Self-advocates in the Opening the Door Working Group emphasized the need to “share the success” and identify the capabilities and contributions of individuals with intellectual disability through dissemination. Explicitly describing inclusive strategies used and the success of those methods in dissemination products may contribute to expanded research inclusion by addressing researcher knowledge gaps. Increasing the accessibility of dissemination (Recommendation 7) may also contribute to expanded understanding of inclusive efforts and successes.

#### Recommendation 2: build community partnerships

Develop and maintain strong community partnerships to build trust within communities and support inclusion efforts. Individuals with intellectual disability are often connected to a network of service providers and community organizations. Developing sustainable and mutually beneficial relationships with community partners can open opportunities to better understand the capacities of individuals with intellectual disabilities and their research priorities. In addition, ongoing partnerships will provide a strong foundation for recruitment of a diverse group of research participants. The historical mistreatment of individuals with disabilities has led some guardians, caregivers, and family to be additionally protective of individuals and cautious about their research participation. This additional layer of protection may require researchers to establish relationships with key people (e.g., advocacy group leaders, care/support providers) to successfully recruit and retain participants with intellectual disability.

#### Recommendation 3: use plain language

Examine language used in all research materials and determine how it can be simplified for accessibility of the wider population. Previous research as well as stakeholders from past and current projects have confirmed that language used by university research teams is often complex, inaccessible, and at times may include offensive terminology. Research teams should use plain language in all materials including recruitment materials and consent forms whenever possible. Adding a glossary to documents when complex language or specific terminology is necessary can increase accessibility. Plain language focuses on presenting complex or technical descriptions in a simple or accessible way. Materials should be clear and concise and presented at the lowest possible reading level. There is not a universally accepted reading level that is considered plain language, however, the Association of University Centers on Disabilities defines plain language as 6th grade reading level or below [52]. Complex language creates a wall between research and the broader population where plain language is a universal support that creates accessibility for all people, including individuals with intellectual disability. An example of a plain language glossary of terms was created as a starting point to support the reearchers in the use of plain language in research (Additional file 1).

#### Recommendation 4: simplify consent (and assent) processes

Increase the accessibility of a research study’s consent and assent forms and procedures. Obtaining informed consent and assent are essential components of ethical research. Consent and assent forms as required by the university ethics review board must include detailed and often technical study information. The accessibility of consent and assent forms can be impacted by the level of technical language used, large amount of text information, and the layout and font size of the form itself. Researchers should create consent and assent forms that are written in plain language, include simple images or diagrams to explain processes and steps when possible, and have succinct descriptions to reduce word count. All consent and assent forms should be formatted according to guidelines for universal accessibility (e.g., Customer Communications Toolkit for the Public Service – A Universal Design Approach [53]) to promote accessibility for all participants.

Many individuals with intellectual disability have assistance with caretaking and decision making (e.g., supported decision-making agreements). It may be necessary for recruitment and consent processes to include additional stakeholders in the individual’s life in order to establish informed consent. In addition, adults with intellectual disability often have legally authorized representatives who make decisions on their behalf. Obtaining informed assent from individuals who have legally authorized representatives in addition to consent from their representative is essential for respectful and meaningful inclusion. Finally, consent processes are poorly understood by the general population and inclusion of legally authorized representatives can be complicated. To support a participant’s understanding of the process, researchers can utilize the “Understanding Consent and Assent in Plain Language” summary (Additional file 2).

#### Recommendation 5: establish research capacity to consent

Assess an individual’s capacity to consent to an individual study rather than their broader cognitive level or IQ. University ethics review boards may require researchers to assess the capacity of an adult with an intellectual disability to be able to provide informed consent. Researchers have frequently used standardized measures of cognitive function to assess consent capacity (e.g., Mini Mental Status Exam [54]). Assessment of an individual’s understanding of the purpose and procedures for an individual study is a more accurate and potentially inclusive method for determining capacity to consent. For example, the National Institutes of Health offers an alternative procedure to ask participants to answer questions to confirm understanding [55]. Answering questions on the purpose, risks, voluntary nature, and participation requirements of the study may provide a more accurate depiction of an individual’s ability to consent to the specific study.

#### Recommendation 6: offer universal supports and adaptations

Offer key supports and adaptations to all participants. Individuals with intellectual disability want to be involved in research. Barriers to inclusion are frequently related to a lack of necessary accessibility, accommodations and supports. Researchers should consider the accessibility of the places they are completing research including physical accessibility (e.g., ramps, elevators, accessible bathrooms) and ease of transportation to location (e.g., on a public transit route, available parking, signage). Accommodations should be available to increase the accessibility of the research procedures and materials (e.g., use of Supports I Need Checklist (Additional file 3), available alternative communication formats, accessible printed materials, assistance offered for reading or writing). Most individuals are capable of participation with the right accommodations in place and individuals should determine the supports they would like to receive. Stakeholders collaboratively developed a list of accommodations that may support the inclusion of individuals with intellectual disability. Use of the Supports I Need Checklist (Additional file 3) can allow researchers to support the self-determination of individual participants and identify accommodations necessary for inclusive participation.

#### Recommendation 7: practice accessible dissemination

Disseminate findings in an accessible way, using simple language and alternative formats. Dissemination of findings are frequently inaccessible to participants with intellectual disability due to the level of complex language used in publications, paywalls to access journal articles, or awareness of the dissemination outcomes of the study. Accessible and alternative dissemination pathways may aid in building trust between communities and researchers and facilitate knowledge sharing with stakeholder communities. Use of alternative formats that are easily accessed by a broad audience (e.g., newsletters, videos, social media postings, and summaries of academic papers) will increase the reach of dissemination as well as the accessibility. Collaborative dissemination between researchers and community partners can be used to support the mutual benefit and sustainability of partnerships as well as reach a relevant stakeholder audience. The Easy-Read Paper Template (Additional file 4) is designed to structure a plain language summary of academic papers for the purpose of disseminating to community and stakeholder audiences.

## Discussion

A history of ongoing health disparities and exclusion from research participation has created a critical need for increased participation of individuals with intellectual disability in health research. Individuals with intellectual disability are important and capable participants in research studies given appropriate opportunities and accommodations. This study identified 11 key barriers to the inclusion of research participants with intellectual disability from comprehensive literature review, consultation with the institutional review board, and a review of relevant federal regulations. A stakeholder working group worked collaboratively to develop seven recommendations for researchers to begin to address identified barriers. The seven recommendations presented in this paper focus on the use of language, communication, and procedures that promote the inclusion of adults with intellectual disability and work to advance health equity in population health research.

The recommendations presented in this paper were intended to facilitate the inclusion of adults with intellectual disability as research participants to increase representation in research samples. The developed products (Additional files 1-4) were created to support researchers’ implementation of the recommendations and facilitate ongoing inclusive research practices. Inclusion as research participants is an important first step to increase research validity and generalizability of findings to broader populations. Representation and identification within research samples will increase the generalizability of samples as well as allow for exploration of the unique needs and health challenges experienced by individuals with intellectual disability. To truly shift the needle on health disparities and create long term changes, individuals with intellectual disability need to be identified in large national public health, clinical, and community samples to allow for measurement of differences and develop strategies for change.

While the recommendations and developed products were designed to increase university research accessibility for adults with intellectual disability, they can apply more broadly to support the inclusion of diverse participants. Building on concepts of Universal Design [53], the recommended changes will provide increased accessibility for many people beyond just individuals with intellectual disability. For example, these same recommendations will increase accessibility of research to individuals of all levels of intellectual ability, even if they do not meet official diagnostic criteria for intellectual disability. They will also make research more accessible to dual-language learners for which English may not be their primary language. Finally, harder-to-reach participants from a variety of socio-economic and educational backgrounds may find that these recommendations make research more accessible. The combination of a universal design approach while still acknowledging that person-centered accommodations may be needed will support full and meaningful participation in research activities.

The current work highlighted that the regulations and procedures created to protect individuals with intellectual disability may inadvertently be creating barriers to their inclusion. Institutional ethics review boards look to legal guidelines to determine policies and language used to guide specific research studies and protect research participants. Federal regulations are infrequently updated, often do not reflect current preferred language, and tend to be deficit focused to justify the need for protection of research participants’ rights and welfare. In addition, regulations are often written in a way that may caution researchers from being more inclusive in their practices by emphasizing a need for additional protective measures to protect participants deemed vulnerable. Changes at the regulatory level could potentially have a larger impact on inclusive research than individual study practices, though both are important.

Recommendations presented above focus on initial strategies to address current barriers to research participation, especially through the recruitment, consent and assent, and enrollment periods of participation. It is important to note that accommodations and increased accessibility are important throughout participation; thus, must be sustained throughout all parts of the research process and not just available at the start of the research study. Many of the recommendations above (e.g., the use of plain language, confirming understanding by asking the participant questions, and providing requested accommodations and supports) can be applied more broadly to the entirety of the research process. Researchers should consider how to increase accessibility and inclusion from enrollment to completion of the study, including in how the research findings are disseminated.

The presented recommendations were created using a stakeholder engagement framework to guide procedures [37]. Similarly, applying a stakeholder engaged framework alongside the recommendations presented could increase the inclusion of co-researching team members with intellectual disability. Advocacy for inclusive research that includes stakeholders throughout the research process has been growing. Large funding organizations, such as the Patient Centered Outcomes Research institute (PCORI), have prioritized the inclusion of stakeholders in the research process [56]. PCORI is the largest public research funder with a focus on community engaged research [56], and has specifically identified intellectual and developmental disability as a research priority [57]. Inclusion of individuals with intellectual disability as co-researchers is increasing, however, it continues to be an uncommon practice [58]. Recent research supports inclusion of individuals with disabiliites within the research team is valuable and feasible [36, 44, 59]. The presented recommendations may offer a starting point that could apply more broadly to increasing the accessibility of all components of the research process to support the inclusion of stakeholders across the research process and variety of studies.

A major strength of the current study was the full inclusion of individuals with intellectual disability in the research process. Specifically, project conceptualization, execution, and development of the recommendations and products presented was driven by collaborating with a stakeholder team. Inclusive research practices allowed the research team to confidently identify barriers and mitigation strategies that may be effective for a wide range of stakeholders, including regulatory agencies, researchers, and individuals with intellectual disabilities. On the other hand, potential limitation of the study was the involvement of researchers, community members, self-advocates with intellectual and developmental disabilities and members of the Institutional Review Board from a single university. Certain topics around supporting research participation that did not arise in the current context of work may be of importance to explore in future work. Additional research will be necessary to build upon the current findings in order to document varying viewpoints and approaches to inclusive research practices.

Researchers should consider application to their own research context and stakeholders when interpreting recommendations for their own use. In addition, implementation of federal regulations across different institutional ethics review boards may impact the applicability of specific recommendations. Thus, university researchers would ideally adapt these recommendations through consultation with their own institutional review board and community of stakeholders. Institutional stakeholder or community advisory boards are becoming more common and can provide consultation for individual projects when ongoing collaboration is not feasible [60, 61]. Ongoing stakeholder collaboration at a university or research group level would allow for adaptations that apply to a specific university and research project context.

## Conclusion

Inclusion of individuals with intellectual disability in health research is essential to address ongoing health disparities. A history of exclusion and significant barriers to participation in research exist for individuals with intellectual disability. The first step to address these barriers is to provide recommendations and tools to facilitate inclusive practices in university health research. Primarily, it is the responsibility of the researcher to open the door to university research as individuals with intellectual disability want to be included, are eager to join research, and are ready to engage [30]. This paper provides an overview of barriers to participation and key recommendations with accompanying products to support inclusion of individuals with intellectual disability as research participants. The provision of these materials must be paired with ongoing education and dissemination to provide education to researchers that have not been inclusive in past practices but focus on health outcomes that are important to individuals with intellectual disability. Future work should build on these recommendations and products to address barriers at the institutional and regulatory level and seek to provide data on successes and challenges to further adapt and refine processes for inclusion.

## Supplementary Information

**Additional file 1.** Appendix A. Plain Language Glossary.

**Additional file 2.** Appendix B. Understanding consent and assent in plain language.

**Additional file 3.** Appendix C. Supports I Need for Research Participation.

**Additional file 4.** Appendix D. Easy Read Article Template.

## Data Availability

Supporting data is not available for publication. Results and conclusions presented in this paper were derived through active working-group participation. Recordings and transcripts from working-group proceedings cannot be made available for publication due to privacy and consent. Additional requests for additional information can be addressed to the corresponding author.
