# Correction: Opening the door to university health research: recommendations for increasing accessibility for individuals with intellectual disability

**DOI:** 10.1186/s12939-022-01790-6

**Published:** 2023-01-11

**Authors:** Brittany M. St. John, Emily Hickey, Edward Kastern, Chad Russell, Tina Russell, Ashley Mathy, Brogan Peterson, Don Wigington, Casey Pellien, Allison Caudill, Libby Hladik, Karla K. Ausderau

**Affiliations:** 1 Department of Kinesiology, Occupational Therapy Program, 2120 Medical Sciences Center, University of Wisconsin at Madison, WI 53706, 1300 University Avenue, Madison, WI USA; 2 Waisman Center, University of Wisconsin, Madison, WI USA; 3 Special Olympics Wisconsin, Madison, WI USA; 4 Institutional Review Board, University of Wisconsin at Madison, Madison, WI USA

**Correction: Int J Equity Health 21, 130 (2022)**

**https://doi.org/10.1186/s12939-022-01730-4**

After publication of this article [1], the authors reported that a wrong Supplementary file was originally published with this article; it has now been replaced with the correct file.

The original article [1] has been corrected.
