# Supplementary material for: Opening the door to university health research: recommendations for increasing accessibility for individuals with intellectual disability
Source: Int J Equity Health. 2022 Sep 10;21:130. doi: 10.1186/s12939-022-01730-4 (PMC9464400; doi:10.1186/s12939-022-01730-4)
Supplement: Supplementary file 1 — Additional file 1. Appendix A. Plain Language Glossary. [file 12939_2022_1730_MOESM1_ESM.pdf]

Plain Language Glossary Example

Advocacy – Sharing your story, thoughts, and opinions to support a cause that promotes a change.

Accommodations – A change in the environment that makes it easier for yourself or others to participate.

Analyze – To organize and understand the detailed information, sometimes called data.

Analysis – What we get from analyzing information.

Assent (for research) – A person choosing that they want to participate in a research study and know what the study is about and all the risks of participating. A person who has someone else who legally makes their decisions gives assent. Legally authorized representatives, or guardians, gives consent for the person participating.

Barriers – Things that make it harder to do something.

Consumer – A person who uses something for personal use.

Consent (for research)– A person choosing that they want to participate in a research study and know what the study is about and all the risks of participating.

Co-researcher – A person who is on the research team who would normally be a participant and not a researcher.

Critical Thinking – Taking time to reflect on or think about something you read or hear.

Curriculum – A program, course, or workshop that teaches us about a specific topic.

Data – Facts or information collected for research analysis.

Disability Awareness – Knowing about and teaching others about what it is like to have a disability.

Empower – To give someone new knowledge or skills so that they can do something new that they couldn't do before.

Findings/Results – The outcome of the research process or the information you find out after doing research.

Health – Your physical, mental, and social well-being.

Health Disparity – A difference in health between two groups that is caused by things that could be prevented (for example: access to health care or discrimination).

Health Research – Research studies that focus on health of certain groups or health of the whole population.

Inclusion – All people being able to participate in all different kinds of activities they choose.

Incentives – Rewards or money that someone gets after doing an activity that helps motivate them (example: money or gift cards).

Participant – A person who takes part in an activity.

Perspectives – A person's unique opinion or what and how they think about an issue.

Policy – Rules or guidelines that governments and organizations follow.

Population – A group of people with something in common.

Process – A series of actions to achieve a goal.

References – Giving another person credit when you use their ideas, especially in a research paper.

Research – The process of asking a question that is important to us, finding the answers, and sharing the information with other people.

Researcher – A person who does scientific research.

Research Team - A group of individuals who work together to answer a specific research question.

Results/Findings – The outcome of the research process or the information you find out after doing research.

Self-Advocate – A person who speaks for themselves and advocates for their needs. They are an expert in the conditions or diagnoses they have.

Survey – A series of questions asked to better understand a group of people or a specific question.

Voluntary – Doing an activity because you want to, not because you have to

Well-being – A state of being physically, mentally, and socially healthy.
