# Supplementary material for: Opening the door to university health research: recommendations for increasing accessibility for individuals with intellectual disability
Source: Int J Equity Health. 2022 Sep 10;21:130. doi: 10.1186/s12939-022-01730-4 (PMC9464400; doi:10.1186/s12939-022-01730-4)
Supplement: Supplementary file 2 — Additional file 2. Appendix B. Understanding consent and assent in plain language. [file 12939_2022_1730_MOESM2_ESM.pdf]

Plain Language Summary of Consent and Assent for Research

What is a Consent or Assent Process?

- When a researcher tells someone about the research study, all the risks, benefits, and what will happen during the study
- The consent and assent process is when a person decides if they want to join the study.
- Each participant must choose to give consent to join a study
- People who have a legal guardian give assent and their guardian would give consent.
- If someone doesn't want to give consent, they cannot be a part of the study

Consent:

- People who are their own guardians give consent
- Guardians can give consent for another person
- Parents can give consent for their child

Assent:

- People who have a legally authorized representative (guardian) give **Assent** and their guardian gives **Consent**

Giving Consent or Assent for a Research study means:

- You understand the study, what you will be doing, and all the risks of joining the study.
- You are choosing if you want to join the study or not.
- If you give your consent, you want to join the study.

Stop Participating When You Want:

You can stop being a part of the research study at any time or for any reason, even after you give consent or assent.

Why we have consent and assent for research:

- To make sure people are safe in the research study
- So that anyone joining knows all the risks and what will happen during the research
- To make sure everyone knows how the information from the study will be protected
- To make sure participants know what to expect
