# Supplementary material for: Opening the door to university health research: recommendations for increasing accessibility for individuals with intellectual disability
Source: Int J Equity Health. 2022 Sep 10;21:130. doi: 10.1186/s12939-022-01730-4 (PMC9464400; doi:10.1186/s12939-022-01730-4)
Supplement: Supplementary file 3 — Additional file 3. Appendix C. Supports I Need for Research Participation. [file 12939_2022_1730_MOESM3_ESM.pdf]

Supports I need For Successful Research Participation

This sheet is for participants to select their preferences and participation support needs.

When communicating with me about this project:
- ☐ Please only contact me
- ☐ Always include this person ____________________________________

To contact me:
- ☐ An email
- ☐ A phone call
- ☐ Text message
- ☐ Other:_________________

Support
- ☐ I do things on my own
- ☐ I prefer to have a support person with me
- ☐ I would like to have a research mentor
- ☐ Other: _________________

I communicate with:
- ☐ My voice
- ☐ Sign Language
- ☐ Facial expressions and body language
- ☐ I need an interpreter
- ☐ Assistive device: _________
- ☐ Other: _________________

Scheduling
- ☐ I need ____ weeks notice
- ☐ I schedule myself
- ☐ I need help with scheduling
- ☐ Other: __________________
- ☐ Additional Reminders:
  - ☐ Day of event
  - ☐ 1 day before
  - ☐ 1 week before
- ☐ I need research materials in advance to review:
  - ☐ 1 day before
  - ☐ 1 week before
  - ☐ Other: _______________

Meeting length
- ☐ 30 minutes or less
- ☐ 1 hour or less
- ☐ Up to 2 hours long
- ☐ I need frequent breaks (every ___ min)
- ☐ Other: ___________________________

I Learn Best With:
- ☐ Talking
- ☐ Video
- ☐ Written Information
- ☐ Pictures
- ☐ Other: _________________

Preference for Printed Materials:
- ☐ Large font
- ☐ Printed paper handouts
- ☐ Simple language
- ☐ Easy-Read summary (short version)
- ☐ Recorded version
- ☐ Pictures instead of words

Paperwork:
- ☐ I do not need help
- ☐ I would like help to **READ**
- ☐ I would like help to **WRITE**

Virtual Meetings and Internet
- ☐ I have my own device for virtual meetings
- ☐ I need a device to join virtual meetings
- ☐ I have access to the internet
- ☐ I need a hotspot or internet access

Rides for Research Activities
- ☐ I don’t need help with rides
- ☐ I will need a ride
- ☐ I would like help planning rides
- ☐ I need extra time to take the bus
- ☐ I would like to join virtually

Anything else you want to share:
