# Supplementary material for: Opening the door to university health research: recommendations for increasing accessibility for individuals with intellectual disability
Source: Int J Equity Health. 2022 Sep 10;21:130. doi: 10.1186/s12939-022-01730-4 (PMC9464400; doi:10.1186/s12939-022-01730-4)
Supplement: Supplementary file 4 — Additional file 4. Appendix D. Easy Read Article Template. [file 12939_2022_1730_MOESM4_ESM.pdf]

EASY-READ ARTICLE SUMMARY

Article title:

**Key points:**	**Glossary:**
**Why this article matters:**	
**Description of the study:**	
**Research questions:**	
**Important results:**	
**How the results will be used:**	

Full article reference:

Instructions for researchers:

Purpose:
The purpose of the easy-read article summary is to facilitate dissemination of research to community and stakeholder audiences in plain language.

General guidelines for completion:
- Use plain language.
- The summary should be in the lowest possible reading level.
- Try to keep the summary under 2 pages.
- Use pictures or a graphic if appropriate.
- Complete sections with bullet points when possible.

Section specific instructions:

Key points
- Highlight the most important information in this section.
- Often this will be a finding or application of the results.

Glossary:
- Define all important research terms in the glossary.
- Define any long or complex word that you feel is necessary to use in the summary.

Why this article matters:
- How does this work move the science forward?
- Describe why the research is important to your audience.
- Why should the community care about this work?

Description of the study:
- Provide a methods overview.
- Briefly describe how data was collected and how the study was completed.
- Include a brief description of participants if relevant.

Research questions:
- Summarize the purpose of the paper and/or research question.

Important results:
- Key findings and most important information from the results section.
- Make sure to highlight results that are relevant to the audience.

How this research will be used:
- What will happen with the results? Who will use them?
- Describe what is relevant for the community or what will come next.
